# Natural Fiber-Reinforced Thermoplastic ENR/PVC Composites as Potential Membrane Technology in Industrial Wastewater Treatment: A Review

**DOI:** 10.3390/polym14122432

**Published:** 2022-06-15

**Authors:** A.S. Norfarhana, R.A. Ilyas, N. Ngadi, Shubham Sharma, Mohamed Mahmoud Sayed, A.S. El-Shafay, A.H. Nordin

**Affiliations:** 1 School of Chemical and Energy Engineering, Faculty of Engineering, Universiti Teknologi Malaysia (UTM), Skudai 81310, Johor, Malaysia; farahfarhana.as@gmail.com (A.N.); norzita@utm.my (N.N.); abuhassannordin@gmail.com (A.N.); 2 Department of Petrochemical Engineering, Politeknik Tun Syed Nasir Syed Ismail, Pagoh Education Hub, Pagoh Muar 84600, Johor, Malaysia; 3 Centre for Advanced Composite Materials (CACM), Universiti Teknologi Malaysia (UTM), Johor Bahru 81310, Johor, Malaysia; 4 Institute of Tropical Forestry and Forest Products, Universiti Putra Malaysia (UPM), Serdang 43400, Selangor, Malaysia; 5 Mechanical Engineering Department, University Center for Research & Development (UCRD), Chandigarh University, Mohali 140413, Punjab, India; shubham543sharma@gmail.com; 6 Department of Mechanical Engineering, IK Gujral Punjab Technical University, Main Campus-Kapurthala, Kapurthala 144603, Punjab, India; 7 Architectural Engineering, Faculty of Engineering and Technology, Future University in Egypt, New Cairo 11845, Egypt; mohamed.mahmoud@fue.edu.eg; 8 Department of Mechanical Engineering, College of Engineering, Prince Sattam bin Abdulaziz University, Alkharj 16273, Saudi Arabia

**Keywords:** rubber-based membrane, natural fiber, filler, adsorbent, ENR/PVC, thermoplastic elastomer, wastewater treatment

## Abstract

Membrane separation processes are prevalent in industrial wastewater treatment because they are more effective than conventional methods at addressing global water issues. Consequently, the ideal membranes with high mechanical strength, thermal characteristics, flux, permeability, porosity, and solute removal capacity must be prepared to aid in the separation process for wastewater treatment. Rubber-based membranes have shown the potential for high mechanical properties in water separation processes to date. In addition, the excellent sustainable practice of natural fibers has attracted great attention from industrial players and researchers for the exploitation of polymer composite membranes to improve the balance between the environment and social and economic concerns. The incorporation of natural fiber in thermoplastic elastomer (TPE) as filler and pore former agent enhances the mechanical properties, and high separation efficiency characteristics of membrane composites are discussed. Furthermore, recent advancements in the fabrication technique of porous membranes affected the membrane’s structure, and the performance of wastewater treatment applications is reviewed.

## 1. Introduction

Most developing countries are confronted with the problem of water pollution, which is a key worry that must be addressed to ensure people’s well-being. Water pollution occurs when there is a change in physical, chemical, and biological properties from its original state that exceeds the limits and standards set as contained in the Water Quality Standard (WQS) that are harmful to living organisms [1]. In general, this pollution is caused by human activities that have adverse effects on the environment, such as health, living resources, ecological systems, and others [2]. One of the main causes of water pollution is due to a permanent source of wastewater from sewage treatment plants and industry [3,4,5,6,7]. It is estimated that a large amount of wastewater production is concentrated in the palm oil and textile industries [8,9,10,11]. Therefore, effective and capable solutions to overcome this pollution problem are very much needed.

Therefore, the water treatment method using membrane technology is seen to be able to overcome the existing problems. Treatment using the membrane method is expected to reduce the presence of contaminants and in turn improve the quality of wastewater [12]. The advantages of this method are that separation can be performed continuously, it requires low energy, it requires no additives, and is easily combined with other separation processes [13,14,15,16]. However, there are still problems regarding the exploration of materials, and the methods used in the manufacture of membranes ideally require high thermal stability, porous surface structure, low-cost, and high wastewater treatment potential. The use of membranes as a medium for wastewater treatment requires the necessary characteristics in terms of mechanical strength, durability, heat resistance, and porosity [17,18,19]. Thus, the preparation of membranes with high mechanical strength, thermal properties, porosity, and solute removal capability has attracted the attention of researchers in industrial wastewater treatment.

**Figure 1:**
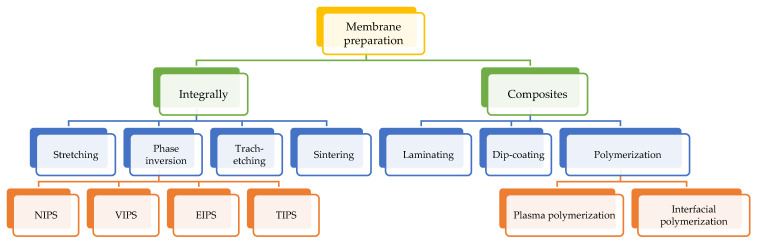
Membrane preparation techniques. Reproduced from [41], Institute of Research and Journals, 2018.

Various types of polymers have been used in the production of membranes for water separation applications, such as cellulose [20,21,22], cellulose derivatives [23,24,25], poly (ethersulfon) (PES) [26,27,28], poly (sulfone) (PSf) [29,30,31], and poly (vinyl chloride) (PVC) [32,33,34,35] as well as the batter. Interestingly, the rubber-based membrane had the potential to be utilized in wastewater treatment applications [36]. According to Tanjung et al. [37], the blending of ENR-50 and PVC could result in a miscible blend due to the creation of hydrogen bonds between the chlorine groups of PVC and the epoxy groups of ENR. The Epoxidized natural rubber/polyvinyl chloride/microcrystalline cellulose (ENR/PVC/MCC) composite membranes for palm oil mill effluent (POME) treatment was prepared [38]. Moreover, the methods employed in membrane preparation will affect the performance of the membrane. According to Siekierka et al. [39], the membrane’s properties depend on the use of appropriate techniques and material modifications to achieve the required structure and morphology for the separation process. Membranes can be made in many ways, including molding, electrospinning, solution casting, sintering, stretching, coating, and phase inversion [40]. Figure 1 shows the membrane preparation technique of asymmetric membranes.

**Figure 2:**
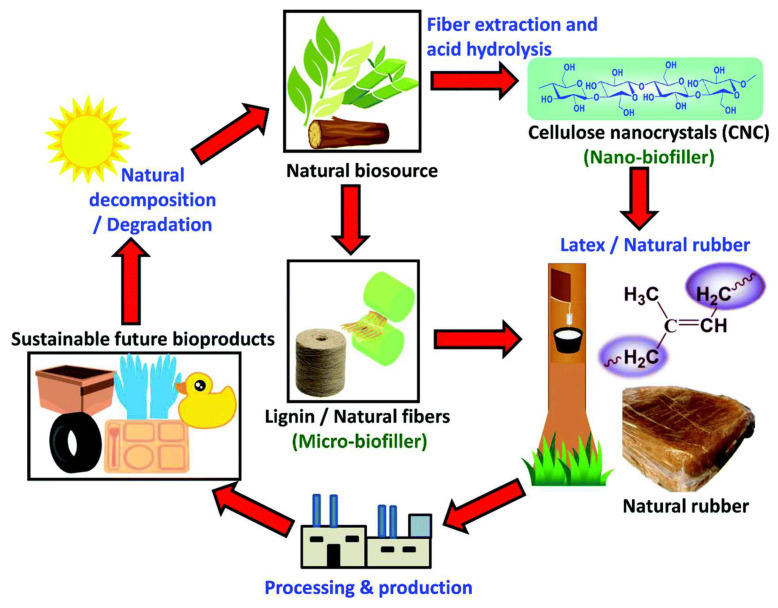
The incorporation of natural fiber in a rubber matrix promising a closed-loop sustainable approach for developing renewable and sustainable rubber. Reproduced with permission from [65]. Copyright 2021 The Royal Society of Chemistry.

Recently, the use of natural fiber as fillers has been gaining the attention of many researchers in the development of polymer composites. Increased awareness regarding the overly worrying problem of agricultural and industrial waste disposal has inspired researchers to exploit these materials [42,43,44]. Natural fibers are abundant resources, low cost, available, biodegradable, not harmful to health, and considered green materials [45,46,47]. Moreover, natural fibers have been widely used as reinforcing fillers in composite materials due to their biodegradability, renewability, and low cost [46]. In addition to overcoming the problem of pollution, the use of natural fiber as a filler has many advantages. Figure 2 shows the incorporation of natural fiber in the rubber matrix, promising a closed-loop sustainable approach for developing renewable and sustainable rubber. Moreover, the addition of fillers to the polymer matrix is to modify the properties of the base polymer and improves its mechanical properties [48,49,50]. Among the natural fillers that are often used are oil palm empty bunch fiber [51,52], coconut fiber [53,54], jute [55,56], pineapple leaves [57,58], sugar palm fiber [59,60] and rice husk [61,62,63,64].

This study aims to review and critically evaluate this growing area of research by exploring the potential of natural fiber as filler and pore former for rubber-based membranes. The fabrication techniques, as well as the effectiveness of the separation process for wastewater treatment applications, have also been discussed.

## 2. Membrane Technology

Membrane technology is developing rapidly, following its use in a variety of applications [66,67,68,69,70]. A membrane is an intermediary between two adjacent phases that acts to control the transport of a substance that has different components [14]. Membranes have different thicknesses and structures according to their application. Based on the shape of the membrane, it consists of symmetrical and asymmetrical membranes. Symmetrical membranes have a homogeneous and relative pore structure, while asymmetrical membranes have a non-homogeneous pore structure. Based on the structure and principle of separation, membranes can be classified into three types, namely, porous membrane, nonporous membrane, and carrier membrane. Membranes consist of natural membranes and synthetic membranes. The natural membrane is a system in the life processes of living beings such as the kidneys. Synthetic membranes are membranes produced by humans that are made from natural materials or synthetic polymers or a mixture of both. Typically, the natural materials used in the production of membranes are cellulose, pulp, and cotton, while synthetic materials include poly (sulfone) (PSf), poly (ethylene glycol) (PEG), and poly (ethylene) (PE), etc. [71,72,73,74,75]. Synthetic membranes are divided into two categories, namely organic (polymer) and inorganic (ceramic) membranes. The use of membranes of the polymer type is more widespread than that of ceramic membranes. In general, all types of polymers can be used in the production of membranes, but the selection of polymers should be appropriate to the method of production of membranes and also its application.

The membrane structure is the most important factor in the principle of separation [76]. An effective membrane in wastewater treatment is a membrane that has pores on its structure to increase the selectivity rate and flux value of the membrane. The principle of separation of porous membranes is based on the difference in particle size of the substances to be separated and the size of the pores on the membrane [76]. Only particles of a certain size can pass through the membrane while the rest will be retained. The size of the pores on the membrane plays an important role in determining the type of membrane separation technique. Table 1 shows the membrane separation technique and the pore size required for its separation application.

Studies of porous membranes produced from porous polymers such as poly (sulfone) (PSf) and poly (vinyl chloride) (PVC) have been conducted by several researchers. Novel TiO_2_ coated functionalized halloysite nanotubes (TiO_2_@HNTs) were embedded with poly(vinyl chloride) ultrafiltration (UF) membranes (PVC/HNTs) for water treatment in the study by Mishra and Mukhopadhyay [77]. The pure water flux of the prepared membrane increased from 127.33 to 212.22 L/m^2^.h for the PVC/HNTs-0 membrane and PVC/HNTs-2 membrane, respectively. The flux recovery ratio for BSA increased from 77.23% (PVC/HNTs-0) to 92.10% (PVC/HNTs-2), and the flux recovery ratio for sewage water went up from 71.42% (PVC/HNTs-0) to 92.16% (PVC/HNTs-2). Bhran et al. [78] fabricated new composite membranes of polyvinyl chloride (PVC) and polyvinylpyrrolidone (PVP) as polymers and tetrahydrofuran (THF) and N-methyl-2-pyrrolidone (NMP) as solvents by using the phase inversion method. The scanning electron microscopy results show that the prepared membranes are smooth and that their pores are distributed evenly across the entire surface and bulk body of the membrane, with no visible cracks. The stress–strain mechanical test demonstrated that the presence of PVP in the prepared membranes improved their mechanical performance. According to the membrane performance results, the salt rejection achieved 98% with high flux. Dong et al. [75] studied the utilization of a bio-derived solvent for nonsolvent-induced phase separation (NIPS) fabrication of polysulfone (Psf) membranes. The pores of Psf/bio-derived solvent membranes resembled sponges, and the membranes exhibited higher water flux values (176.0 ± 8.8 LMH) as well as slightly higher solute rejection (99.0 ± 0.5%).

Polysulfone (PSf) membranes are generally favored for water treatment due to their high thermal stability and excellent chemical resistance [74]. However, the filtration capacity of the polysulfone membrane is limited due to low water flux and poor antifouling ability, both of which are caused by the membranes’ low surface hydrophilicity. In 2019, Nguyen et al. [74] blended graphene oxide (GO) or graphene oxide-titanium dioxide (GO-TiO_2_) with a polysulfone matrix to improve hydrophilic and antifouling properties using the phase inversion method. Experiments have shown that graphene oxide can be used to make stable membranes. Then, by lowering the water contact angle values, the surface of these membranes becomes hydrophilic. This increases the permeability and water flux of methylene blue from the aqueous feed solution, which makes the membrane more resistant to fouling. Huang et al. [73] prepared a series of polysulfone membranes with different pore structures using electrochemical impedance spectroscopy (EIS). The impact of electrolyte concentration on the impedance spectrum of polysulfone membranes was then investigated in depth.

Sun et al. [71] integrated and implemented a novel, mussel-inspired, sticky catechol-functionalized poly (ethylene glycol) (Cate-PEG) as an additive to modify the hydrophobic poly (vinylidene fluoride) (PVDF) ultrafiltration (UF) membrane to reduce the leakage of poly (ethylene glycol) (PEG) from the membrane matrix for practical water treatment applications. Surface segregation allowed the Cate-PEG polymer to migrate from the matrix onto the membrane surface and internal pores, resulting in a hydrophilic membrane. Moreover, the PVDF/Cate-PEG UF membrane demonstrated a high-water flux, good BSA rejection, and satisfactory antifouling performance following BSA solution cycling tests. An electrospun polyvinylidene fluoride (PVDF) nanofiber-supported TFC membrane with high performance has been successfully manufactured [79]. Negatively charged electrospun polyacrylic acid (PAA) nanofibers were deposited on electrospun PVDF nanofibers to form a support layer of PVDF and PAA nanofibers. This result indicates more hydrophilic support than the plain PVDF nanofiber support. The PVDF-LbL TFC membrane produced enhanced hydrophilicity and porosity without giving up mechanical strength. Consequently, it exhibited a high pure water permeability and low structural parameter values of 4.12 L/m^2^.h.bar and 221 µm, respectively, which were significantly superior to those of commercial FO membrane.

Mansourizadeh et al. [80] also reported on the production and characterization of PSf porous ring fiber membranes using the phase inversion method. The resulting membrane has a high porosity with the addition of glycerol as a pore-generating agent. The results of the study also found that the addition of glycerol concentration up to 5 wt% has narrowed the pore diameter, thereby increasing the porosity of the membrane. However, the opposite occurs when the glycerol concentration exceeds 5 wt%.

Chinpa et al. [81] prepared and characterized a porous asymmetric membrane of PVC/poly (methyl methacrylate-co-methacrylic acid) (P (MMA-MAA)) through a phase inversion technique. The addition of P (MMA-MAA) to the PVC solution produced larger pores on the membrane surface. The size of structured pores, such as radius and membrane porosity, increased with increasing P (MMA-MAA) composition, thereby increasing the permeability and flux of bovine serum albumin (BSA). However, the increase in pore size on the membrane has lowered the values of tensile strength and elongation at the breaking point.

In a study conducted by Lin et al. [82], porous PMMA/Na+-montmorillonite (MMT) cation-exchange membranes were produced for cationic dye adsorption [82]. Srivastava et al. [83] have studied the capability of modified poly (vinylidene fluoride) (PVDF) membranes for ultrafiltration of textile wastewater [83]. The effect of the addition of Styrene-acrylonitrile (SAN) into PVDF was studied. SAN was added to the PVDF from 0 to 100 wt%. The study found that the number of pores increased with the addition of SAN and in turn increased the water flux. The modified PVDF membrane successfully removed 97% of the congo red dye (CR) and over 70% of the five reactive black dyes (RB5) from the original solution.

### 2.1. Membrane Fabrication Techniques

Moreover, an important point in the process of membrane separation is the nature of the membrane itself. The properties of the membrane depend on the use of appropriate methods and material modifications to obtain the appropriate structure and morphology for the separation process [39]. Various methods are used to produce membranes such as molding [84,85], solution casting [86,87], sintering [88,89], stretching [90], coating [91,92] and phase inversion [40,93,94,95]. The solution casting techniques, phase inversion techniques, and electrospinning are the most frequently used techniques for membrane production. The solution casting method is a process in which a solution is poured into a mold and allowed to solidify at room temperature [87]. The shape of the membrane is influenced by the shape of the mold used. Pore formation is expected to result during the solvent evaporation process during the drying process.

#### 2.1.1. Phase Inversion Method

**Figure 3:**
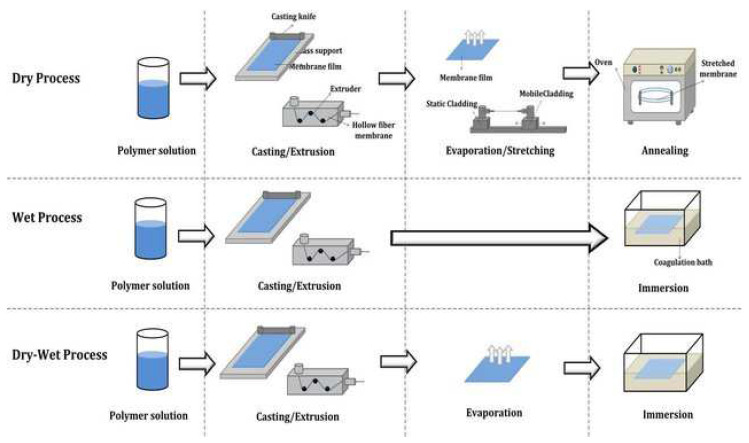
Diagrammatic representation of membrane fabrication by phase inversion process. Reproduced with permission from [108]. Copyright 2019 Penerbit UTM Press.

Lately, many researchers have chosen the phase inversion approach for the manufacture of porous membranes. Figure 3 shows the diagrammatic representation of membrane fabrication by phase inversion process. The phase inversion process is the process of exchange of a polymer from the liquid phase to the solid phase that occurs under controlled conditions [96]. Phase separation occurs when the solvent and nonsolvent phases change when the solution is immersed in an agglomeration container [97]. This process produces a porous layer on the membrane surface [81,98]. The resulting pore structure depends on several parameters, such as the composition, additives, and temperature of the agglomeration container. The morphological properties of the membrane are strongly influenced by the properties of the material and its processing conditions. The phase inversion method is the most effective method for producing porous membranes [99]. This method is the most popular and widely used technique in membrane preparation [100]. It refers to a technique of exchange of a poured polymer solution from a liquid phase (polymer solution) to a solid (macromolecular network or gel) [101,102]. During this phase inversion process, a thermodynamic equilibrium occurs and causes the polymer solution to separate into two phases namely the polymer-rich phase and the polymer-less phase. The polymer-rich phase will form a membrane matrix, while the polymer-less phase will form pores. This method can be carried out in several ways, as shown in Table 2.

The phase inversion method is the most widely used in the preparation of porous membranes. Feng et al. [103] made a study on the preparation and characterization of membranes from poly (vinylidene fluoride-co-tetrafluoroethylene) using the phase inversion method [103]. Dimethylacetamide solvent (DMAc) and trimethyl phosphate pore-forming agent (TMP) were used. The effects of solution composition, agglomeration process time, and agglomeration container temperature on the structure of poly membrane (vinylidene fluoride-co-tetrafluoroethylene) were studied. The resulting membrane morphology showed that the number of pores on the membrane increased with increasing TMP composition. The presence of pores on the membrane results in higher flux values compared to PVDF membranes without TMP. High flux values have increased membrane permeability and selection rates [104,105]. Yang et al. [106] conducted a study on the preparation of microbial membranes from cellulose/glucomannan conjugation (KMG) in an aqueous NaOH/thiourea solution using the phase inversion technique. The polymer solution was poured on the surface of a glass plate and spread using a casting knife to produce a membrane with a thickness of 0.24 mm and then immersed in calcium chloride (CaCl_2_) for 10 min. The resulting membrane has micro-sized pores, and the pore size increases with increasing KGM composition. A portion of the KGM is extracted out into the immersion of running water, causing pores to form on the membrane.

A polyethersulfone (PES)/microcrystalline cellulose (MCC) composite membrane for humic acid (HA) removal in water was made by Nazri et al. [107] using the phase inversion method. A rheological study showed that MCC-containing casting solutions increased viscosity, affecting the composite membrane’s pore structure. Comparatively, composite membranes have larger surface pores, an elongated finger-like structure, and sponge-like pores. The water contact angle and pure water flux of the composite membranes indicated that their hydrophilicity had increased. However, the permeability of composite membranes began to decrease at 3 wt% MCC and above. The natural organic matter removal experiments were performed with humic acid (HA) as the surface water pollutant. The hydrophobic HA rejection was greatly increased by the enhanced hydrophilic PES/MCC composite membrane through interaction between hydrophobic and hydrophilic groups and pore size exclusion.

A study on the production of asymmetric cellulose acetate with the addition of poly (ethylene glycol) (PEG) was once conducted by Saljoughi et al. [109]. In this study, the polymer was dissolved in a 1-methyl-2-pyrrolidone (NMP) solvent. The polymer solution is poured and spread on a glass plate using a casting knife. The dispersed film is immersed into a nonsolvent that produces a polymer precipitate which is a membrane. Morphological studies show that pores can be produced in large numbers when the CA concentration decreases, the PEG concentration and temperature increase, and the flux value of pure water also increases. Mahendran et al. [110] prepared ultrafiltration membranes from cellulose acetate (CA)/poly (sulfone sulfonate) (SPS) and cellulose acetate (CA)/epoxy resin (ER) blends using solution mixing and phase inversion techniques [110]. The effect of SPS and ER polymer material composition on the flux value and water permeability rate of the CA membrane was studied. The concentration of PEG additive on the ultrafiltration properties of the membrane was also studied. The results of the study found that the polymer composition and the concentration of additives in the polymer solution have influenced the properties of the membrane, such as membrane resistance and water content.

#### 2.1.2. Electrospinning Method

**Figure 4:**
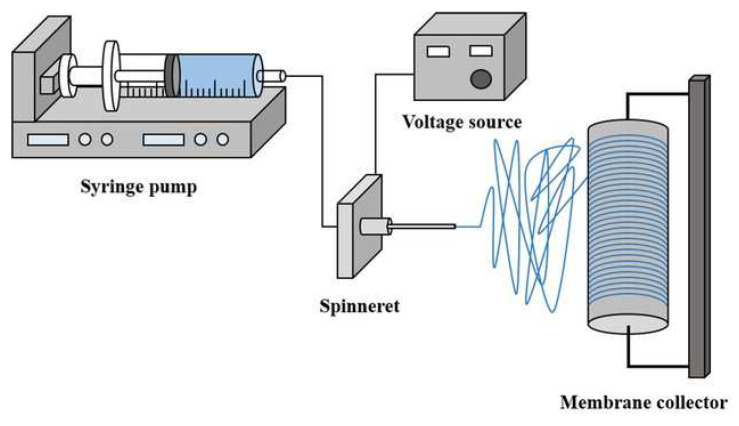
The electrospinning setup for fabrication of nanofibers. Reproduced from [79], MDPI, 2018.

Electrospinning is a versatile method for making nonwoven nanofibrous membranes with a submicronic-interconnected pore-like structure that can be used in a wide range of applications at a low cost [111]. Electrospun polymer nanofibers have emerged as one of the most encouraging and evolving engineered materials for membrane synthesis due to their extremely high porosity, high permeate flux and selectivity, excellent physicochemical stability, and tunable properties [112]. Nanofibers made with this unique electrospinning process have a large surface area compared to nanofibers made with other spinning processes. In a typical electrospinning process, a polymeric solution in a syringe is exposed to a high DC electric voltage. The syringe needle is connected to the positive terminal of the DC supply, while the negative terminal is connected to a collector plate. Beyond a certain electric field (threshold voltage), the repulsive electrostatic forces overcome the surface tension of the polymeric solution, and a loaded flow of the polymer solutions is ejected from the tip of the Taylor cone at the syringe needle in the form of nonwoven fibers. Solvent evaporation depends on the distance between needle tip and collector, solution vapor pressure, temperature, and humidity in the spinning chamber [111]. Figure 4 depicts a schematic representation of an electrospinning process for nanofibers fabrication. Ren et al. [113] conducted experiments utilizing an electrospinning technique to produce gas diffusion layers (e-GDLs) composed of nanosized carbon fibers with a nanoscale pore structure. In addition, vapor deposition of Dow Corning Sylgard 184 was used to apply a hydrophobic coating to the e-GDL to increase its hydrophobicity. The e-GDL has excellent elastic deformability, which can effectively mitigate the irreversible damage caused by the pre-tightening force during the stack assembly process, thereby improving the durability and lifetime of PEMFCs.

### 2.2. Applications of Membrane Technology in Wastewater Treatment

Industrial wastewater means wastewater or sewage water that has been used in industrial activities. Wastewater from various industries will produce wastewater that has a variety of organic substances. Most industrial industries in Malaysia produce wastewater that is liquid and is still rich in organic matter that is easily decomposed. Excessive disposal will cause odor pollution and water pollution that disrupts the human life system. Therefore, all industrial operators who dispose of wastewater have been required by the authorities to first treat industrial wastewater before it is discharged into rivers to avoid environmental pollution.

Malaysia is well-known for its palm oil sector, and it is one of the world’s leading producers of palm oil goods, accounting for around 41% of worldwide palm oil production [114,115]. However, the production of palm oil has led to the discharge of Palm Oil Mill Effluent (POME), which is very much resulting in serious water pollution [100]. The POME produced has a high rate of biochemical oxygen demand (BOD), chemical oxygen demand (COD), total suspended solids (TSS), and high turbidity. It has been reported that POME production for 2005 was 44.88 million metric tonnes, and of this amount, BOD was estimated at 1.122 million tonnes, which is equivalent to the pollution produced by 61,479,500 people (with an average per resident producing 0.05 kg of BOD per day).

Various treatment methods, including physical, chemical, biological, or a combination thereof, have been used to treat wastewater from this industry [116,117,118,119,120,121,122,123]. However, these methods are still seen to be less effective and less efficient because the resulting wastewater has various compositions and is difficult to classify in general [124,125]. The chemical treatment process is a surprisingly rapid method of treating water but is often seen as less effective because the cost of purchasing the chemical is quite high and harmful to the environment [126]. Meanwhile, the biological wastewater treatment process generally uses a large area and a lot of energy [127,128]. This situation will be a problem for industries located in places with narrow areas. In addition, the biological treatment process requires a long time for the process of decomposition of its organic matter before being released into the river. This will cause an increasingly serious problem of odor pollution and will disrupt the daily lives of locals [3,129].

Apart from the palm oil industry, the textile manufacturing industry is also one of the most important industries in Malaysia. Indeed, the textile manufacturing industry has long been practiced in this country and is very famous on the east coast peninsular of Malaysia. The growth of the textile industry in Malaysia has increased the rate of its wastewater production every year. According to Hassan et al. [130], the textile industry contributes 22 percent of total wastewater generation in Malaysia. Wastewater from the textile industry contains a lot of dye content that is difficult to decompose depending on the nature of the chemical, its molecular size, metals, and salts. According to Yuan et al. [131], industrial wastewater contains various types of chemicals, such as enzymes, sodas, dyes, salts, and acids, that will cause serious environmental pollution. Textile wastewater produces dyes and suspended solids, and high COD values will cause allergies, cancer, and skin irritation if left untreated [132]. Therefore, the separate treatment of organic materials, as well as the decolorization of these dyes, must be performed according to standards before being released into the river to overcome the problem of pollution. For wastewater from the textile manufacturing industry, the most commonly used treatment methods are ozone treatment, biological oxidation, chemical agglomeration, and adsorption [83]. Nevertheless, the treatment of wastewater containing dyes has posed serious problems in its decolorization process [124]. High pH values and salt concentrations, as well as complex chemical structures, require more effective and efficient treatment than existing methods [133]. Therefore, to eliminate the problem, one of the other technologies that can be used to clean up industrial wastewater is membrane technology.

Membrane technology in water and wastewater treatment is a physical separation process that separates larger components from smaller ones. Various types of membrane separation techniques are categorized based on the type of driving force applied, the type and configuration of the membrane, and its removal capability [15]. Membrane processes are used in drinking water and wastewater treatment systems, such as in desalination processes, removal of organic matter, removal of colors, particles, and others [16,117]. Today, membrane technology is used in industrial wastewater processing and treatment industries [134]. This technology has been around for the past 25 years, and in recent times, the process has undergone rapid development. Industrial wastewater treatment using membrane technology has proven that this technology has a high potential to overcome the problem of environmental pollution [12]. Membranes can filter contaminated organic matter and then obtain and recycle clear water for the processing plant [135]. Treatment methods using membrane technology are gaining attention because these alternative methods provide more efficient treatment methods, require minimal energy, and do not require the addition of chemicals into the waste system. Thus, membrane separation technology is one of the potential technologies to treat industrial wastewater without disturbing the environmental balance [136].

The quality of wastewater from the palm oil industry (POME) that has been treated using membrane technology is much better than water treated by conventional methods in terms of the level of clarity and odor [136]. The results of his research prove that the treatment process using this membrane technology takes a maximum of only three days compared to the existing treatments (aerobic and anaerobic processes), which take from 80 to 120 days. Membrane technology has great potential in POME treatment systems. This is due to its high ability to separate contaminants from POME as well as recover high-quality water. Sulaiman and Ling [137] studied the potential of bare fiber membranes with MWCO ranging from 30 to 100 K in POME treatment [137]. Studies show that the use of these membranes has successfully reduced the values of COD, TSS, TKN, and nitrogen-ammonia by 97.66%, 98.00%, 53.85%, and 61.91%, respectively.

As for wastewater from the textile manufacturing industry, Chakraborty et al. [138] reported that textile wastewater treated using membrane technology can reduce wastewater production and, in turn, reduce its treatment costs [138]. Textile industry wastewater treatment using various types of membrane processes proves this technology has the potential to overcome pollution problems [138]. Several researchers [139,140,141] have reported that membrane technology is highly effective in the treatment of textile wastewater.

Laqbaqbi et al. [142] applied the direct contact membrane distillation for textile wastewater treatment using a flat-sheet polyvinylidene fluoride (PVDF) membrane. The results demonstrated that high separation factors (α) were achieved (>99.73%), demonstrating substantially less wetting and penetration of the dyes across the membrane pores. Karim et al. [143] synthesized biobased composite membranes for water purification by freeze-drying and packing cellulose nanocrystals (CNCs) in a chitosan matrix. Positively charged dyes, such as Victoria Blue 2B, Methyl Violet 2B, and Rhodamine 6G, were effectively removed 98%, 84%, and 70% of the time by the membranes after 24 h of contact. Fersi et al. [144] treated textile wastewater using microfiltration membrane (MF), ultrafiltration (UF), and nanofiltration (NF) separately and showed more than 90% of color, turbidity, TDS, and COD were eliminated [141]. Suksaroj et al. [145] reported that nanofiltration is one of the membrane technologies that can remove the color, COD, and salinity of textile wastewater [145]. Karkooti et al. [146] developed advanced nanocomposite membranes employing graphene nanoribbons and nanosheets for water purification. The development of polymeric membranes may provide an effective solution to improve water recycling. Four different graphene oxide (GO) derivatives were incorporated into a polyethersulfone (PES) matrix using a nonsolvent induced phase separation (NIPS) method. The GO derivatives used have different shapes (nanosheets vs. nanoribbons) and oxidation states (C/O = 1.05–8.01), with the potential to improve water flux and reduce membrane fouling via controlled pore size, hydrophilicity, and surface charge. All graphene-based nanocomposite membranes exhibited superior water flux and organic matter rejection in comparison to the unmodified PES membrane. The fouling measurements revealed that fouling was impeded due to the improved surface properties of the membrane. Longitudinally unzipped graphene oxide nanoribbons (GONR-L) loaded at 0.1 wt% provided the highest water flux (70 LMH at 60 psi), organic matter rejection (59%), and antifouling properties (30%) improvement compared to the pristine PES membrane.

Overall, it may be said that membrane technology emerged for the efficient separation of wastewater. Abouzeid et al. [147] suggest that water purification membranes or filter technology are good ways to filter water because they are very effective and do not create any secondary pollutants.

## 3. Rubber-Based Membrane

The rubber-based membrane has been utilized for a variety of sustainable industrial applications, including pervaporation, gas separation, and water treatment. Bicy et al. [148] investigated the interfacial tuning and designer morphologies of microporous membranes made from nanocomposites of polypropylene and natural rubber. Alquraish et al. [149] use the latex phase blending and crosslinking technique to make nitrile butadiene rubber-graphene oxide (NBR-GO) membranes. This new way of membrane fabrication is good for the environment and makes membranes that separate oil and water. GO was discovered to change the surface morphology of the NBR matrix by introducing creases and folds on its surface, increasing the membrane’s permeation flux and rejection rate efficiency. The membrane containing 2.0 pphr GO can permeate 7688.54 L/m^2^.h water at an operating pressure of 0.3 bar, while the oil percentage removal is 94.89%. As GO loading increases from 0.5 to 2.0 pphr, fouling on the membrane surface increases from 45.03% to 87.96%. Nevertheless, chemical backwashing might recover 100 percent of the membrane’s performance.

### 3.1. Epoxidized Natural Rubber Elastomer (ENR)

**Figure 5:**
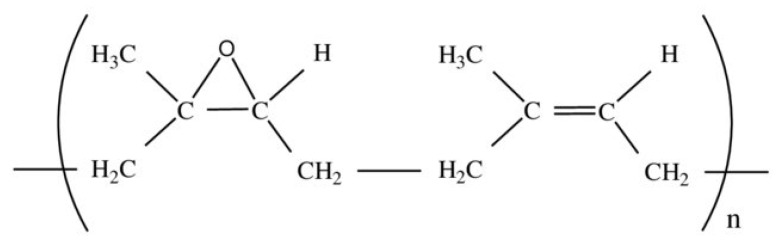
Epoxidized Natural Rubber (ENR) molecular structure. Reproduced from [155], IOP Publishing Ltd, 2018.

Natural rubber (NR) is derived from the latex of a rubber tree (*Hevea Brasileansis),* which consists of more than 98% polyisoprene [150]. Epoxidized Natural Rubber (ENR) is natural rubber (NR) to which epoxide units have been added to the double bond chain, as shown in Figure 5 [151]. This process is better known as an oxidation process that involves a simple reaction and usually uses inexpensive reagents [152], such as acetic peroxide, which are formed in situ with formic acid and hydrogen peroxide [151]. ENR has better properties than NR in terms of oil resistance, wet grip, high absorption properties, and gas permeability [153]. In industry, there are three grades of ENR used based on the degree of epoxidation on its chain structure, namely ENR-10 (10% mole epoxy), ENR-25 (25% mole epoxy), and ENR-50 (50% mole epoxy). Table 3 shows the properties of the three ENR grades [154].

ENR-50 is the most widely used ENR grade because it has a high polarity compared to the others. ENR-50 was used as a base material in various studies due to its unique properties [156,157]. Among them is ENR-50, which is elastic, oil-resistant, has high abrasion resistance, is hydrophilic [158], and has high tensile properties [154]. The presence of high epoxy groups on the NR chain allows ENR to interact better with fillers and result in more crosslinking with other polar polymers. The addition of fillers to the ENR chain can also improve its mechanical properties. Today, among the fillers often used in industry are carbon black (CB) [159,160], calcium carbonate (CaCO_3_) [161,162], and silica (SiO_2_) [163,164,165,166]. A study conducted by Kim and Eom [167] has proved that there is a bond between ENR with silica from rice husk (RH) flour that has high thermal stability when mixed. Ahmad et al. [168] conducted a study on the effect of filler addition (carbon black N110, SiO_2,_ and CaCO_3_) on the mechanical properties of NR/LLDPE blend composites. The composite dough is prepared using a Haake Rheomix machine. Fillers are added to the batter by 10 to 60 *w*/*v*. It was found that the mechanical and physical properties of the mixtures depended on how the filler was described (particle sizes, structure, and surface properties). Carbon black-filled NR/LLDPE blends have a higher bound rubber content than silica-filled NR/LLDPE blends, and calcium carbonate has the lowest bound rubber content. As expected, the rubber–filler interaction is greater for carbon black-filled NR/LLDPE blends than for other fillers, where the carbon black particles interact strongly with rubber chains, resulting in the formation of chemical bonds. In addition, silica has a lot of hydroxyl groups on its surface, which makes them interact strongly with each other. Intermolecular hydrogen bonds between hydroxyl groups on the surface of silica are extremely strong; consequently, they can agglomerate tightly, which results in the formation of filler networking. The study conducted by Ismail et al. [163] showed that there is an increase in the mechanical properties of rubber when silica is added to the natural rubber matrix. This increase is directly proportional to the diffusion of silica in the rubber matrix and the increase in crosslink bond density through silica agglomeration.

### 3.2. Poly(vinyl chloride) (PVC) Thermoplastic

**Figure 6:**
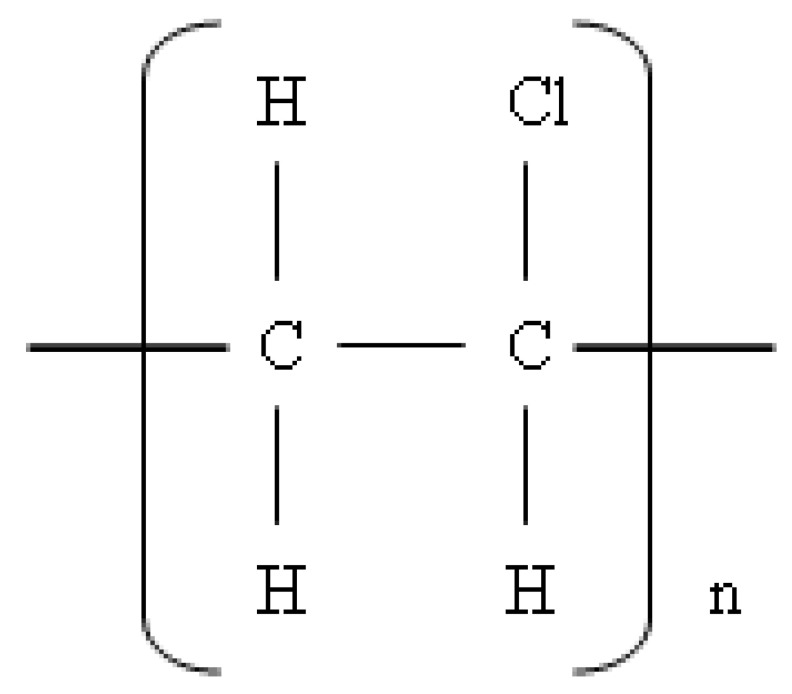
Poly(vinyl chloride) (PVC) structure.

Poly(vinyl chloride) (PVC) is a type of thermoplastic polymer formed from a straight and long bonding chain and composed of vinyl chloride monomers, as shown in Figure 6. PVC is a form of polymer that has a wide range of applications in the engineering, medical device, packaging, and construction industries, among others [169,170].

PVC has a specific density of 1350 kg/m^3^ and is an inexpensive, durable, and recyclable thermoplastic. In addition, PVC can produce materials that have high stress and can provide elastic and flexible properties with the addition of plasticizers [171]. Therefore, PVC is widely used in the market as a basic material for manufacturing goods, such as pipes and toys. However, the disadvantage of PVC is that it is rigid and brittle and has very limited thermal stability. Therefore, PVC blends with elastomers such as ENR can overcome its disadvantages [172].

### 3.3. Thermoplastic Elastomer ENR/PVC Blends

The properties of a polymeric material can be improved by mixing two or more different polymers. Hanafi Ismail [173] reported that the mixing of two or more different polymers provided better and more unique properties than the original properties of each of the materials. Various types of polymer blends are gaining attention nowadays, such as elastomer–elastomer, thermoplastic–thermoplastic and thermoplastic–elastomer (TPE) blends [174]. Elastomer is a type of polymer that is elastic, while thermoplastic is a type of plastic that can be melted repeatedly. The combination of these two polymers, namely TPE, will produce a material that has properties that can be enhanced from the original polymer.

TPE is a new class material that combines the vulcanized properties of rubber with the ability to simplify thermoplastic processes [175]. Elastomers and thermoplastics help in their mixing for the strengthening of the material. Softer elastomers can help thermoplastics increase the impact resistance and toughness of materials, while thermoplastics can increase the rigidity of elastomeric materials [176]. According to Bhowmick [177], TPE blends are one of the blends that have attractive properties because their processing characteristics are the same as those of thermoplastics and their technical properties are similar to vulcanized elastomers [177]. When rigid thermoplastic properties are mixed with low rubber properties, the modulus value will produce a material that has better mechanical properties. Among the TPEs that have been produced are blends of elastomers such as natural rubber (NR), nitrile rubber (NBR), and epoxidized natural rubber (ENR) with thermoplastics such as poly (vinyl chloride) (PVC), poly (ethylene) (PE) and poly (propylene) (PP) that aim to produce TPEs with specific specifications.

**Figure 7:**
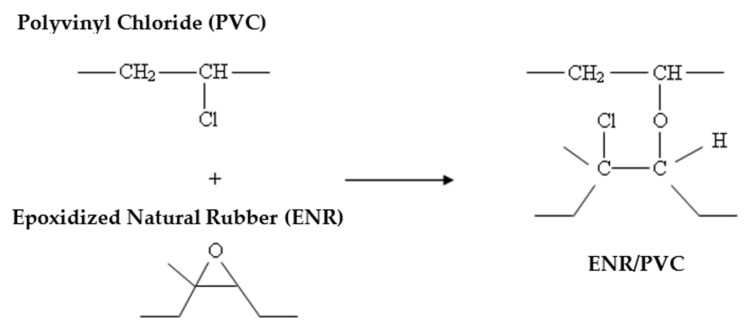
Crosslinking reactions between ENR and PVC. Redrawn from [183], Wiley, 1991.

Epoxidized natural rubber (ENR) is a renewable material and is a hydrocarbon polymer that is compatible with PVC [178]. ENR is a flexible polymer that has high resistance to oil and high mechanical properties [179], while PVC is a rigid and brittle polymer [180]. The blend of ENR and PVC forms a thermoplastic elastomer (TPE), which has high mechanical, elastic, flexible, and processing ability properties [172]. PVC is expected to impart high tensile strength, and good chemical resistance, whereas ENR has good tear strength and acts as a permanent plasticizer to PVC. TPE can be produced by blending ENR with synthetic thermoplastics, where the compatibility of ENR with other polymers is determined by the polarity of the ENR molecule [181]. The blending of ENR enhanced compatible polymers to produce strong TPE. The strength of this mixture was constructed based on the strong volcanic adhesion interaction between ENR and PVC. Ibrahim & Dahlan [182] described the interaction of volcanic adhesions or crosslinking reactions formed between ENR and PVC. Figure 7 shows the crosslinking reaction between these two polymers [183].

Based on the reaction proposed by Ramesh and De [183], the epoxy group on the ENR can act as a proton acceptor, and this allows the occurrence of specific interactions with chlorine on PVC [152]. When ENR and PVC are mixed at high temperatures, the decomposition of the C–Cl groups on PVC increases and produces hydrochloric acid (HCl). At the same time, the opening of the epoxy group ring on the ENR becomes furan. This hydrochloric acid will react with the epoxy group on the ENR and form a chlorohydrin group that acts as a reactive site [184].

Ratnam et al. reported that the tensile strength of ENR-50 increased when blended with PVC [185]. Ramesh and De [186] reported that ENR/PVC thermoplastic elastomer blends had the properties of oil resistance, abrasion resistance, and high modulus values. According to Ratnam and Zaman [185], a blend of ENR-50 together with polyvinyl chloride (PVC) will form a thermoplastic elastomer that is compatible with any reaction ratio [185]. Varughese and his colleagues [184] conducted a study on the mechanical properties of ENR-50/PVC blends at different compositions [184]. Rigid PVC becomes more flexible when blended with ENR. However, the tensile strength, tear strength, and hardness decreased due to the elastomeric properties of the ENR.

The physical properties of TPE depend on the mixing method, mix composition, morphology, and cross-bonding or maturation in the polymer mix. The most popular TPE blending method is melt mixing due to its very simple and easy process. Processing conditions, temperature, and mixing time are the parameters that determine the degree of interaction and mixing between the components in the TPE mixture. Nasir et al. [187] conducted a study to determine the optimal mixing conditions for ENR/PVC blends with the melt mixing method using the Brabender Plasticoder. The study found that the composition of the mixture between rubber and thermoplastic will affect the mixture temperature and rotor speed. In polymers, when ENR is more dominant than PVC, high temperatures and low rotor speeds are required to produce a compatible TPE. Studies have found that the mixing of thermoplastic phases such as PVC with ENR can improve the physical properties of the mixture, but when PVC is more dominant, the TPE mixture cannot maintain its tensile strength. The dough becomes more brittle and hard, causing a decrease in mechanical properties at a certain aging temperature.

Blending ENR with PVC can produce a compatible TPE that has both rubber and thermoplastic properties. However, the production of membranes from elastomeric materials is not porous [188,189]. Therefore, ENR/PVC blends as membranes require pores for industrial wastewater treatment applications. The addition of fillers can help improve the mechanical properties of ETP, in turn, acting as a pore generating agent on the membrane so that it can be applied for industrial wastewater treatment.

The addition of reinforcing filler to thin-film composites has improved mechanical properties [48]. By including reinforcing fillers in the polymer matrix, thin-film selectivity and strength can be increased [190]. Ray et al. [191] reported that filler loading natural rubber (NR) membranes showed better toluene selectivities than unfilled membranes. The addition of fillers may increase the surface area and mechanical strength of the membrane. Table 4 shows that the addition of filler in the ENR/PVC matrix improves the properties of composites. Increasing the use of natural fiber-reinforced composites attracted much attention in the past few years [192,193]. Agricultural fillers (such as kenaf, pineapple, rubberwood, and palm oil empty fruit bunch) have been used to improve the material properties of polymer composites because of their low cost, low density, high specific strength, modulus, environmental friendliness, and renewable nature [164]. Normally, fiber type fillers improve tensile strength because the fibers are able to support stresses transferred from the polymer [194].

## 4. Natural Fiber-Reinforced Polymeric Membrane

**Figure 8:**
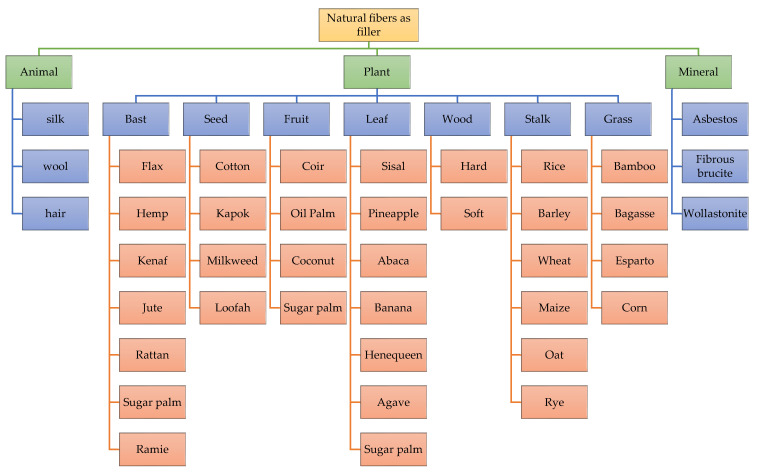
Classification of natural fibers as filler for polymer composites.

To date, global industries have thought about using natural fibers as an alternative to synthetic materials as one of the components in composites due to renewable nature and good marketing appeal in composite manufacturing industries [201,202]. The exceptional characteristics of natural fibers, such as low cost, low density, recyclability, biodegradability, and resource and abundance sustainability, make them the preferred material [203]. Natural fibers that come from either animals or plants can be used as fillers in polymer composites [204,205,206]. Fillers are fine solids added to synthetic resins, rubber, or paints to improve their mechanical properties without altering the molecular structure of the polymer. Figure 8 shows natural fibers that are used to strengthen the matrix to improve and enhance the physical, thermal and mechanical properties of materials as well as reduce costs [207,208,209,210,211,212,213,214,215,216,217]. According to Bicy et al. [148], nanofiller shape and localization have a substantial impact on the membrane’s properties and porosity [218,219,220].

In 2020, Mark et al. [207] investigated the effects of filler loading on the mechanical and morphological properties of carbonized coconut shell particle-reinforced polypropylene composites. The coconut shells were carbonized, pulverized, and sieved into four particle sizes: 63, 150, 300, and 425 µm, with loadings of 0, 10, 20, 30, and 40 wt% for each particle size. The filler exhibited improved yield strength, tensile strength, tensile modulus, flexural strength, flexural modulus, and hardness of polypropylene as filler loading increased. The filler exhibited improved mechanical properties in the composites. Due to strong interfacial adhesion, SEM revealed a positive filler–matrix interaction. The incorporation of more filler resulted in the formation of more spherulite-producing nuclei, the diminution of pore sizes, and an improvement in particle size distribution and mechanical properties. The study conducted by Ismail et al. [154] is related to the effect of filling oil palm empty bunch fibers in three size ranges, namely 270–500, 180–270, and 75–180 µm on the dispersion and its interaction with the polymer matrix. Studies have found that rubber composites with smaller-sized fiber powders show high mechanical properties. This is because smaller-sized fillers have a large surface area, which in turn, increases the interaction of the rubber matrix on the filler surface.

In general, the strengthening ability of a filler is influenced by three main characteristics, namely particle size and surface area, surface shape and structure, and the activity and chemical properties of the surface [49]. This will have an impact on important properties of the composite, such as processing ability, density, and aging performance [221]. When fillers are added to the tensile strength value matrix, the modulus and hardness increase with a decrease in filler particle size. Fillers such as silica (SiO_2_) and carbon black (CB) can act as reinforcers if they have a small particle size and a large surface area. The addition of a small-sized filler that is in the nanometer range will increase the surface area of the particles, which causes the filler to play its role more effectively in its dispersion in the polymer matrix more evenly [222,223,224,225,226,227]. The incorporation of fine particles produces a large surface area and will disperse more evenly or homogeneously in the polymer matrix, further increasing the tensile strength of the composite [207]. This is because the addition of fillers into the polymer matrix has improved the mechanical properties of the membrane [228,229]. Furthermore, the addition of a smaller-sized filler will increase the surface area of the filler causing the filler to be dispersed more evenly in the matrix [230]. The results of the study showed that the improvement of the mechanical properties of the material depends on the surface interaction and adhesion between the filler and the matrix, as well as the uniform distribution of the filler in the composite.

Moreover, the efficiency of fillers in improving the mechanical properties of materials is highly dependent on the interface interactions and adhesion of fillers and matrices [231,232]. Premalal et al. [190] conducted a study related to the addition of rice husk (RH) and talcum fillers in polypropylene (PP). The results showed that the addition of RH powder into the matrix had increased the value of modulus, elongation at the breaking point, and tensile strength but lower than talc due to weak interface interaction between RH powder and the PP matrix compared to talc.

Recently, the use of organic or natural fillers has been gaining the attention of many researchers due to the increasing awareness related to the problem of agricultural waste disposal, which is worrying and causes pollution. The use of natural fillers to replace inorganic fillers has many advantages, including unlimited resources, low cost, availability, easily performed chemical and mechanical processing, and not endangering health [154,233]. Among the natural fillers that are often used are wood fiber, oil palm empty bunch fiber, coconut fiber, jute, pineapple leaves, henequen waste, and rice husk. Some researchers have reported the advantages of using natural fillers in thermoplastic matrices due to their unique properties, such as being readily available, cheap, low density, easily biodegradable, and environmentally friendly. In addition, the use of natural filler in the polymer matrix has significant benefits because the strength and toughness of the matrix can be increased [234]. Referring to Torres and Cubillas [235], lignocellulose fiber reinforced plastic materials have higher mechanical properties, are environmentally friendly, and reduce costs.

### 4.1. Matrix Filler

The biodegradability of natural fibers is deemed the most significant and intriguing aspect of their use in polymeric materials [236]. When fillers are added to the polymer matrix, the toughness, elasticity, and tensile strength all go up [49]. Rice husk (RH) is a natural filler that is a cellulose fiber that can be used in composite manufacturing panels. These natural fibers are assessed as an environmentally friendly, low-density source and an inexpensive and readily available alternative and can be used as fillers to improve the mechanical properties of a composite [237]. Based on some of the properties of these natural fillers, many researchers have taken the initiative to apply their use in the field of composites [173,188,238,239,240]. Table 5 shows the main compositions of the RH powder.

In 1975, Haxo and Mehta [156] reported that rice husk had 34–44% cellulose, 23–30% lignin, 13–39% ash, and 8–15% moisture. Rice husk (RH) is a source of high cellulose and even silica, which can improve the mechanical properties of the material. According to Handayani [241], several studies have shown that rice husk ash contains a lot of silica content of 94–96%. Open-fired RH contains more silica and has a high potential as a filler in thermoplastics to replace synthetic fillers such as carbon black [157]. In a study conducted by Ahmad et al. [242], the addition of RH and clay into the matrix of high-density liquid/liquid natural rubber/poly (ethylene) (NR/LNR/HDPE), NR/HDPE, and HDPE has increased the value of the composite tensile modulus. This is because RH has improved the stiffness properties of the composite material by filling in the empty spaces in the matrix. According to [64], the addition of RH improved thermal stability, modulus, and the number of pores in the ENR/PVC membrane. The ENR/PVC membranes with 5 wt% and 10 wt% RH loading had pores on the surface, which improved the water absorption, flux, and permeability of the membranes, according to SEM pictures.

In 2001, Hanafi Ismail et al. [164] reported that the addition of rice husk ash in NR/LLDPE could improve the tensile modulus and hardness properties of the composite. The addition of RH into the matrix has reduced the movement of the polymer chains, thereby improving the stiffness properties of the material and resulting in composites that have better thermal stability. Yang et al. [238] have studied the effect of RH addition on poly (propylene) (PP) matrices. RH was added from 10 to 40 wt%, and composites were produced using melt blending techniques. The study found that the addition of RH increased the value of tensile modulus but decreased the tensile strength of the composite. In general, this is due to an incompatibility between hydrophilic lignocellulose fillers with hydrophobic matrices. Poor surface interaction and adhesion between the filler and the matrix led to a decrease in the tensile strength of the composite. In addition, the addition of RH into the matrix has complicated the movement of the polymer chains and improved the stiffness (modulus) properties of the composite.

Weaknesses of interface and adhesion interactions between RH particles (hydrophilic) and polymer matrices (hydrophobic) are a major problem in the production of composites with these natural fillers [243]. The moisture of these natural fillers can cause the mechanical properties of the resulting composites to be degraded. The fibers have a lignin layer that makes it difficult for them to interact well when blended with a polymer matrix. Through the study of Jamil et al. [244], it was shown that natural rubber blended with high-density polyethylene (NR/HDPE) filled with rice husk and liquid natural rubber (LNR) as adapters could change the composite properties in terms of mechanical properties, thermal properties, and homogeneity of the resulting dough. Based on the study, the tensile strength of the composite was found to decrease with the addition of RH in the matrix, but the tensile modulus was found to increase with the presence of RH. Poor adhesion between the matrix and the filler causes the distribution of the filler to the whole matrix to be inhomogeneous, and the occurrence of particle clumping causes the properties of the composite to become weak, thereby lowering the tensile strength of the composite [245]. However, this problem can be overcome by the addition of LNR in the composite matrix because LNR has reduced the hydrophilic properties of RH and increased the interaction of the filler interface, and the matrix in turn shows an increase in the mechanical properties of the composite.

Therefore, the addition of a stabilizer or surface treatment on the natural fibers improves the compatibility with the polymer matrix [246]. Among the treatments that are often used is the use of gamma radiation, treatment with isocyanates, silane, peroxides, and alkali [247]. Alkaline treatment using sodium hydroxide (NaOH) is one of the widely used treatments by researchers aimed at improving the interface surface interaction and adhesion between RH and matrix [248]. Moreover, this surface modification treatment aims to improve the adsorption properties of RH [249]. In the treatment of lignocellulose fibers, NaOH acts as a lignin binding agent because lignin is easily soluble in NaOH and at the same time removes all impurities and oil residue present on the surface of cellulose fibers. NaOH treatment improved the hydrophilic properties of lignocellulose fibers. This is due to the removal of lignin, hemicellulose, and fat layers as well as increased porosity or active surface area on RH [250]. Therefore, lignocellulose fibers more easily absorb water from an environment that is also hydrophilic. The increase in such characteristics is due to the increase in more active hydroxyl (–OH) terminals on the surface of lignocellulose fibers after treatment is performed [248,251]. Several researchers have reported that RHs that have been treated with NaOH can improve the mechanical properties and adsorption properties of untreated materials [248,250,251].

### 4.2. Pollutant Adsorbent

Natural fiber emerged as a promising adsorbent material for pollutant removal due to its availability and abundance of hydroxyl groups. RH has potential as a dye and heavy metal adsorbent for wastewater treatment [252,253], in addition to being a filler in composites. Akhtar et al. [254] conducted a study related to the adsorption potential of RH to remove 2,4-dichlorophenol (DCP) from an aqueous solution. The effects of RH thermal treatment, stirring time, amount of adsorbent, pH of the solution, and amount of DCP absorbed were studied for the adsorption of DCP in an aqueous solution. The study found that thermally treated RH could improve the removal of DCP from an aqueous solution more effectively than chemically treated RH. Therefore, the thermally treated RH is used for the next stage and applied for wastewater application. After 10 min of stirring time with an increase in RH composition of 0.025–0.1 g, the adsorption percentages of DCP increased up to 97% and 66%, respectively. For industrial wastewater applications, the results show that RH has eliminated DCP by 99 ± 0.2%. Studies show that RH is a cheap and easily available adsorbent and can effectively remove DCP from industrial wastewater.

Ajmal et al. [255] conducted studies on the removal of cadmium (II) in an aqueous solution using RH. The process of removal of this organic matter depends on the contact time, solution concentration, pH, and temperature. The results of the study found that RH treated with phosphate had improved the removal of cadmium (II) from wastewater. Katal et al. [256] conducted a study on the adsorption of nitrate from aqueous solutions and industrial wastewater using modified RH. The effects of contact time, amount of adsorbent, pH of the solution, and temperature of solution on nitrate adsorption in aqueous solution were studied. The highest adsorption capacity was at pH 7, 90 min, and 0.4 g/100 mL, where the percentage of nitrate removal increased to 93.4%. For industrial wastewater applications, studies show that the modified RH removed nitrate at a concentration of 34.7 ppm by 91.8%. This indicates that RH has high potential as an adsorbent and removes nitrates in industrial wastewater.

The use of RH as an adsorbent for dyes such as methylene blue (MB), congo red (CR), and brown carmine (IC) has been studied by many researchers. Chakraborty et al. [249] conducted a study on the adsorption of purple crystal dye (CV) in an aqueous solution by RH treated with NaOH [249]. The results of the study found that NaOH-modified RH has the potential to remove dyes through the adsorption process based on several parameters such as pH, amount of adsorbent, temperature, and initial concentration. Therefore, the use of this natural resource as an adsorbent is one of the alternatives to treating industrial wastewater because RH is an unlimited source, readily available, and cheap.

## 5. Conclusions and Future Perspective

Dyes, saturated salts, heavy metals, organic compounds, and oil emulsions represent a substantial danger to water supplies, which is a major global problem. Membrane technology for contaminant removal is becoming prominent since it provides more efficient treatment methods, requires less energy, and does not require the addition of chemicals to the waste stream. Rubber-based membranes have elastic, flexible, ductile, and robust properties and are commonly employed in the pervaporation and gas separation process. The use of rubber-based membrane for various industrial separation processes has been explored, but few works have addressed a rubber-based membrane in water separation applications. It is because the rubber-based membrane has a dense structure and has no pores for water permeation. Hence, the addition of natural fibers as filler and pore former was able to improve the thermal stability, mechanical characteristics, morphology, and performance of ENR/PVC membrane, as well as their potential for use in the water separation process. Furthermore, the use of natural filler in a polymer matrix is consistent with the polymer’s excellent properties for a variety of applications. The excellent properties of rice husk (RH), which is a rich source of cellulose, and even silica can improve the mechanical properties of a material. RH also can be used as an adsorbent to adsorb dyes from wastewater treatment. The potential of RH as filler, pore former, and adsorbent in rubber-based membrane enhanced the separation process of wastewater. In addition, the fabrication method of the membrane also affects the morphology and properties of the membrane and enhances the water permeability of wastewater. Thus, the incorporation of RH into the ENR/PVC matrix resulted in the formation of a porous membrane with the potential for wastewater treatment applications.

## Figures and Tables

**Table 1:** Membrane separation technique and pore size.

Membrane Separation Technique	Pore Size
Mikrofiltration (MF)	0.04–100 µm
Ultrafiltration (UF)	0.1–1 µm
Nanofiltration (NF)	100 Å–0.001 Å
Reverse Osmosis (RO)	1 Å–10 Å

**Table 2:** Membrane synthesis by using phase separation technique.

Technique	Principle
Thermally-induced phase separation (TIPS)	- This method is based on the fact that when the temperature is lowered, the solvent quality usually decreases. The solvent is removed by extraction, evaporation, or freeze-drying after demixing.
Air-casting of a polymer solution	- A volatile solvent and a less volatile nonsolvent are mixed to dissolve the polymer. The polymer’s solubility diminishes as the solvent evaporates, allowing phase separation to occur.
Precipitation from the vapor phase	- Phase separation of the polymer solution is caused by the entrance of nonsolvent vapor into the solution during this process.
Immersion precipitation	- A thin layer of polymer solution is cast on support or extruded through a die, then immersed in a nonsolvent bath. Precipitation can happen when the polymer solution’s excellent solvent is replaced by a nonsolvent.

**Table 3:** ENR properties depend on grade.

	ENR-10	ENR-25	ENR-50
Glass Transition Temperature, T_g_ (°C)	−60	−45	−20
Specific Gravity	0.94	0.97	1.03
Mooney Viscosity, M_L, 1 + 4_ (100 °C)	90	110	140

**Table 4:** Filler for ENR/PVC matrix composites.

Filler	Fabrication Techniques	Properties	Applications	Ref.
Oil palm empty fruit bunch (OPEFB)	Electron-beam irradation	Tensile strength, Young’s modulus, and gel content increase with a concurrent reduction in the elongation at break (Eb) of the composites.	Composite material	[195]
Oil palm empty fruit bunch (OPEFB)	Melt blending	Young’s modulus, hardness, and flexural modulus of the PVC/ ENR blend increase with the increase in OPEFB loading	Composite material	[196]
Rubber-wood	Melt blending	Flexural modulus, Young’s modulus and hardness increased with the RW loading. The impact strength, Ts and Eb decrease with the increase in RW loading	Composite material	[194]
Titnium dioxide (TiO_2_)	Melt blending, radiation	Good distribution of TiO_2_ in the PVC/ENR blends matrix	Composite material	[197]
Pineapple leaves fiber cellulose	Solution blending, casting technique, phase inversion method	Number of pores increased with the addition of cellulose. Decoloration of palm oil mill effluent after treated by ENR/PVC/Cell-20% and ENR/PVC/Cell-g-PMMA-10% membranes.	Composite material	[36]
Rice husk powder	Solution blending, casting technique, phase inversion method	Relative humidity (RH) reduces tensile strength and increases the tensile modulus. The number of pores increased with the increasing wt% of RH.	Water permeation	[198]
Silica	Solution blending, casting technique, phase inversion method	Thermal and mechanical stability of the membranes improved with the incorporation of silica.CO_2_ and N_2_ gas permeation of silica-filled membranes increased with increasing silica content	Gas permeation	[189]
Silica	Solution blending, casting technique, phase inversion method	Silica as pore former. Mechanical properties of the membrane improved by the addition of silica. COD and BOD showed a reduction of 44% and 38.3%, respectively, after POME	POME treatment	[199]
Magnesium Oxide, MgO	Solution blending, casting technique, phase inversion method	Pores developed as fillers were introduced to the membrane.Permeability values of CO_2_ and N_2_ increased with the addition of MgO.	Gas permeation	[200]
Microcrystalline Cellulose, MCC	Solution blending technique	Chemical oxygen demand (COD), biochemical oxygen demand (BOD) and total suspended solid (TSS) were reduced to 99.9%, 70.3%, and 16.9%, respectively.	POME treatment	[38]

**Table 5:** Main rice husk (RH) composition.

Element	Percent (%)
Cellulose	25–35
Hemicellulose	18–21
Lignin	26–31
Silica	15–17
Solute	2–5
Humidity	7.5

## Data Availability

Not applicable.
